# Neurobiological effects of phospholipids *in vitro*: Relevance to stress-related disorders

**DOI:** 10.1016/j.ynstr.2020.100252

**Published:** 2020-09-15

**Authors:** Francisco Donoso, Marina Schverer, Kieran Rea, Matteo M. Pusceddu, Bernard L. Roy, Timothy G. Dinan, John F. Cryan, Harriët Schellekens

**Affiliations:** a APC Microbiome Ireland, University College Cork, Cork, Ireland; b Department of Psychiatry & Neurobehavioural Science, University College Cork, Cork, Ireland; c Department of Anatomy & Neuroscience, University College Cork, Cork, Ireland; d Cremo SA, Fribourg, Switzerland

**Keywords:** Phospholipids, Neuroprotection, Neurodevelopment, Stress

## Abstract

Nutrition is a crucial component for maintenance of brain function and mental health. Accumulating evidence suggests that certain molecular compounds derived from diet can exert neuroprotective effects against chronic stress, and moreover improve important neuronal processes vulnerable to the stress response, such as plasticity and neurogenesis. Phospholipids are naturally occurring amphipathic molecules with promising potential to promote brain health. However, it is unclear whether phospholipids are able to modulate neuronal function directly under a stress-related context. In this study, we investigate the neuroprotective effects of phosphatidylcholine (PC), lysophosphatidylcholine (LPC), phosphatidylserine (PS), phosphatidylethanolamine (PE), phosphatidylinositol (PI), phosphatidylglycerol (PG), phosphatidic acid (PA), sphingomyelin (SM) and cardiolipin (CL) against corticosterone (CORT)-induced cytotoxicity in primary cultured rat cortical neurons. In addition, we examine their capacity to modulate proliferation and differentiation of hippocampal neural progenitor cells (NPCs).

We show that PS, PG and PE can reverse CORT-induced cytotoxicity and neuronal depletion in cortical cells. On the other hand, phospholipid exposure was unable to prevent the decrease of *Bdnf* expression produced by CORT. Interestingly, PS was able to increase hippocampal NPCs neurosphere size, and PE elicited a significant increase in astrocytic differentiation in hippocampal NPCs. Together, these results indicate that specific phospholipids protect cortical cells against CORT-induced cytotoxicity and improve proliferation and astrocytic differentiation in hippocampal NPCs, suggesting potential implications on neurodevelopmental and neuroprotective pathways relevant for stress-related disorders.

## 1 Introduction

It has long been known that neuronal plasticity can affect cognitive processes and emotions, and impairments in neuronal signalling directly impacts on mental health (Wohleb, 2016; Birkel, 2017). The relative abundance of specific nutrients from dietary sources can positively modulate neuronal function and can offer protective effects against environmental and physiological factors (Gómez-Pinilla, 2008). Indeed, dietary nutritional components have been demonstrated to be crucial in several brain processes including brain development, neuroplasticity and neurogenesis (Georgieff, 2007). In particular, evidence suggests that optimal nutrition is important for the maintenance of adult hippocampal neurogenesis (An et al., 2008; Dyall et al., 2010; Heberden, 2016; Poulose et al., 2017), the process where new neurons are produced from neural progenitor cells (NPCs) in the hippocampus (Eriksson et al., 1998). The process of neurogenesis is most pronounced during embryonic development, but there is evidence that it persists into adulthood with relevant implications for hippocampal-dependent cognitive function throughout (Kohman and Rhodes, 2013). On the other hand, contradicting reports indicate that the very existence of this neuronal process can still be a subject for debate (Lee and Thuret, 2018).

On the other hand, the emerging and compelling evidence for nutrition as a crucial factor in the high prevalence and incidence of mental disorders suggests that changes in diet are a viable strategy for improving mental health and treatment of stress-related psychiatric disorders including anxiety and depression (Sanchez-Villegas and Martínez-González, 2013; Jacka et al., 2014; Opie et al., 2015; Adan et al., 2019). Stress-related mental disorders are associated with alterations in the hypothalamic-pituitary-adrenal (HPA) axis, which can be activated in response to stress leading to a release of glucocorticoids into the circulation (Sapolsky et al., 2000). Although these steroid hormones are critically involved in the homeostatic regulation of metabolism, chronic exposure in the central nervous system (CNS) to glucocorticoids has been associated to deleterious effects on neuronal structure and function (Mitra and Sapolsky, 2008).

Although all nutrients are important for neuronal function, some appear to have a prime role in terms of neuronal growth and development, as well as neuroprotection during vulnerable stages involving high exposure to stressful events (Calabrese et al., 2008; Keunen et al., 2014). Based on these observations, the identification of key nutrients and nutritional components that can boost and protect mental health by modulating neuronal processes may represent novel therapeutic strategies against stress-related neuropsychiatric disorders.

In this regard, phospholipids are specific nutrients consisting of lipids with a hydrophilic head formed by a phosphate group and two hydrophobic tails (Kullenberg et al., 2012), with promising potential to promote resilience to stress and improve age-related cognitive decline (Schverer et al., 2020). Naturally occurring phospholipids can be found in plant and animal food sources, including eggs, organ and lean meats, fish, shellfish, cereal grains, oilseeds and milk (Cohn et al., 2010), and are considered to be important components of nutrition, since studies have demonstrated that phospholipid-enriched diets significantly impact behaviour (Kullenberg et al., 2012). Indeed, phospholipid supplementations have shown significant improvements in aged individuals in terms of cognitive performance (Crook et al., 1991), and attenuated the negative effects of stress in young adult subjects (Benton et al., 2001). However, it is not clear whether the positive effects of phospholipids on stress-related behaviour are linked to direct modulation of neuronal function or indirect systemic responses.

Currently, there is a lack of studies looking at the beneficial effects of phospholipids on mental health and disease, and more research is needed to understand their therapeutic anti-stress effects. In this study, we hypothesise that specific phospholipids may possess neurobiological properties and may exert beneficial effects against stress in cellular models. An individual screening rather that a combination of phospholipids will provide relevant information for elucidating potential mechanisms. In addition, to our knowledge, no other studies have investigated individual members of the phospholipid family for their potential to exert neurobiological effects. Thus, the purpose of the present study was to explore through an *in vitro* approach the implications of phospholipids on neurobiological processes vulnerable to stress. A panel of nine individual phospholipids was assessed for their neuroprotective capacity against CORT-induced cytotoxicity in neurons derived from the whole brain cortex. In addition, we tested the effect of this panel on proliferation and neural differentiation in hippocampal NPC derived neurospheres, considering the implications of this brain area in neurodevelopment and neurogenesis (Anacker and Hen, 2017; Fares et al., 2019) as well as in stress-related psychopathologies (Levone et al., 2015). Hippocampal NPCs forming neurospheres are a valuable tool for understanding the physiology of adult CNS stem cells and neurogenesis, as well as for isolating neurobiological pathways associated with stress (Reynolds and Rietze, 2005; Kino, 2015; O'Leime et al., 2018). Together, findings from this study provide new insights into the potential neurobiological effects of specific phospholipids *in vitro* and add important direction to future research for the prevention and treatment of stress-related neuropsychiatric disorders.

## 2 Materials and methods

### 2.1 Chemicals and reagents

Dulbecco's modified Eagle's medium (DMEM), penicillin/streptomycin, D-glucose, foetal bovine serum (FBS), L-glutamine, Trypsin, DNase I, corticosterone (CORT), Poly-L-Lysine, 3-(4,5-Dimethyl-2-thiazolyl)-2,5-diphenyl-2H-tetrazolium bromide (MTT), fibroblast growth factor (FGF), epidermal growth factor (EGF), and dimethyl sulfoxide were purchased from Sigma. Phosphatidylcholine (PC; from egg yolk), lysophosphatidylcholine (LPC; from egg yolk), phosphatidylserine (PS; from bovine brain), phosphatidylethanolamine (PE; from egg yolk), phosphatidylinositol (PI; from soy bean), phosphatidylglycerol (PG; from egg yolk), phosphatidic acid (PA; from egg yolk), sphingomyelin (SM; from egg yolk) and cardiolipin (CL; from bovine heart) were obtained in their respective salts from Sigma. B-27 supplement was obtained from Thermo Fisher Scientific.

### 2.2 Animals

All procedures on live animals were performed under licence from the Government of Ireland Department of Health (B100/3774) in accordance with National and European Union directive 2010/63/EU, with prior ethical approval by University College Cork (AEEC #2012/045). Experiments were conducted in accordance with guidelines established by University College Cork's Animal Welfare Body.

### 2.3 Primary culturing of post-natal day 1 rat cortical neurons

Mixed neuron and astrocyte cultures were prepared as described previously (Pusceddu et al., 2016; Donoso et al., 2019). Briefly, post-natal day (PND) 1 Sprague Dawley male rats were sacrificed and cerebral cortices dissected. Cortical tissue was dissociated into a cellular suspension using trypsin (0.25%; 37 °C) and physical trituration with a glass Pasteur pipette in warmed DMEM-F12 with 10% FBS and 100 μg/mL DNase I. Cell suspension was passed through 40 μm strainer and then centrifuged at 1000 rpm for 10 min at room temperature. Cells were re-suspended in warmed culture media (DMEM-F12 supplemented with B-27, 1% FBS, 100 U/mL penicillin, 100 μg/mL streptomycin, L-glutamine 2 mM and D-glucose 55 mM), and then cultured at 37 °C with 5% CO_2_.

### 2.4 Isolation and culturing of rat hippocampal neural progenitor cells

Hippocampal cultures containing neural stem cells (NSC) and neural progenitor cells (NPC) were obtained as described previously (O'Leime et al., 2018). Briefly, hippocampal tissue was dissected from embryonic day (E) 18 rats, and then gently triturated with a glass Pasteur pipette in warmed proliferative media (DMEM-F12 supplemented with 10 ng/mL EGF and 10 ng/mL FGF, B-27, 100 U/mL penicillin, 100 μg/mL streptomycin, 2 mM L-glutamine and 33 mM D-glucose). In these proliferative conditions, cells were allowed to form floating neurospheres for 7 days *in vitro* (DIV). For differentiation studies, untreated neurospheres were dissociated to a single cell suspension after proliferation for 7 DIV, and seeded at a density of 4 × 10^4^ cells/poly-L-lysine-coated 13 mm glass coverslip in 24-well tissue culture plates using differentiation media (DMEM-F12 supplemented with B-27, 1% FBS, 100 U/mL penicillin, 100 μg/mL streptomycin, 2 mM L-glutamine and 33 mM D-glucose), and then cultured at 37 °C with 5% CO_2_.

### 2.5 Cell treatment

**Fig. 1:**
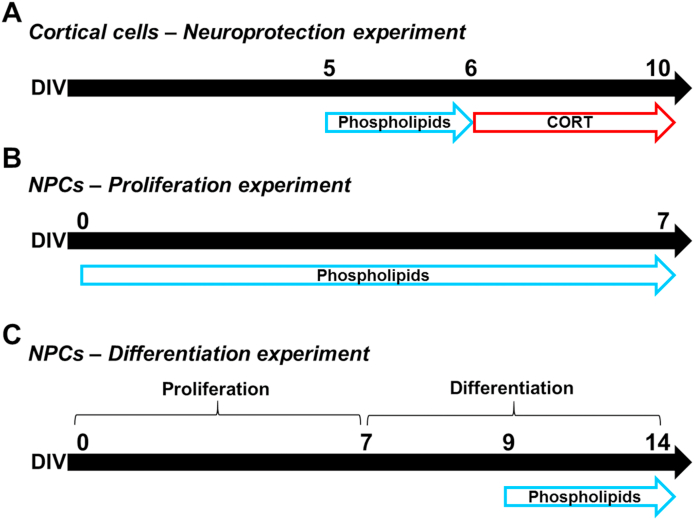
**Schematic representation of the in vitro experiments performed**. **(A)** Neuroprotection experiment. Cortical cells were incubated with various concentrations of phospholipids for 24 h from DIV5 to DIV6, then the media was replaced with 200 μM CORT and kept until DIV10. **(B)** Proliferation experiment. NPCs were exposed to phospholipids and allowed to form neurospheres under proliferative conditions from DIV0 to DIV7. **(C)** Differentiation experiment. NPCs-forming neurospheres were incubated in proliferative conditions from DIV0 to DIV7 without any stimulus. At DIV7 neurospheres were disaggregated and incubated with differentiation media. Phospholipids were added to the media from DIV9 to DIV14. Differences in optimal treatment duration in these experiments were established according to previous results (data not shown).

A phospholipid concentration range of 0.05–30 μg/mL was added to cortical cells at DIV5 and maintained for 24 h. We have previously established that treating cortical cells from any day before DIV5 and longer than 24 h disturbs the neuronal/glial development and attachment in the culture plate. Then the media was replaced with fresh media containing 200 μM CORT at DIV6 and kept for 96 h (Fig. 1A) to induce cell death as previously reported (Pusceddu et al., 2016; Donoso et al., 2019). This dose of CORT was established as the minimum concentration able to induce significant changes in cortical cells. For NPC-derived neurospheres at proliferative conditions, phospholipids (0.05–30 μg/mL) were added at DIV0 and maintained until DIV7 (Fig. 1B). For differentiation studies, untreated NPCs were treated with phospholipids (0.05–30 μg/mL) for five days (Fig. 1C). The doses of phospholipids were chosen based on previous publications, which reported functional activity *in vitro* (Arakawa et al., 1991; Kalvodova et al., 2005; Treede et al., 2007).

### 2.6 Cell viability measurement

Cell viability was determined by the MTT assay which is based on mitochondrial dehydrogenase activity of viable cells as previously described (Mosmann, 1983). Briefly, cortical cells or NPCs were cultured at 4 × 10^4^ cells per well in 24 well-plates. After treatment, medium was removed and replaced with fresh culture medium containing MTT (500 μg/mL), and then incubated at 37 °C for 3 h. Then, in order to dissolve the formazan produced after MTT reaction 100 μL of dimethyl sulfoxide were added to each well. The absorbance values were measured by spectrophotometry at 570 nm with a microplate reader (BioTek Synergy HT). The results were expressed as a percentage of vehicle control group.

### 2.7 Immunocytochemistry

Cellular staining for neuronal (βIII-tubulin) and astrocytic (GFAP) markers, was assessed as through immunofluorescence detection as previously described (Pusceddu et al., 2016; Donoso et al., 2019). Briefly, cortical cells and NPCs were fixed with ice-cold methanol for 10 min and then blocked overnight in 5% horse serum at 4 °C. Then the cells were incubated overnight at 4 °C in primary antibody solution (mouse anti-βIII-tubulin 1:300, Promega; rabbit anti-GFAP 1:300, Dako). The following day, cells were washed and incubated with secondary antibody solution (Alexa Fluor 594 donkey anti-mouse 1:2000, Thermo Fisher; Alexa Fluor 488 donkey anti-rabbit 1:2000, Thermo Fisher) for 1 h at room temperature. Nuclear staining was performed with Hoechst 33258 (Sigma) for 5 min. Cells then were counted in 5 fields of view for each coverslip and analysed using an Olympus BX53 upright fluorescence microscope. Between 50 and 70 cells were counted in each field of view.

### 2.8 Analysis of NPC neurosphere growth

NPCs were cultured in 6-well plates at 4 × 10^5^ cells per well under proliferative conditions. Selected doses of phospholipids were added at DIV0. Neurospheres were viewed under an inverted Olympus IX70 microscope at DIV2, DIV4 and DIV7 using bright field imaging. At least 5 images per condition were captured, and the neurosphere diameter was quantified using ImageJ 1.51j8 software. Each condition was repeated in three different experiments. At DIV0 NPCs received selected doses of phospholipids for 7 days, and micrographs were taken at DIV2, DIV4 and DIV7. The different phospholipids were distributed randomly across two experiments performed on 2 different days to be able to accommodate the high number of experimental conditions. In the first experiment, PS, PG, PA, and CL were tested (Fig. 5A); while PC, PI, PE, and SM were examined in the second experiment (Fig. 5C).

### 2.9 Quantitative RT-PCR

Total RNA from primary cortical cells was isolated using the High Pure RNA isolation kit (Roche). Briefly, cells were seeded at density of 1.5 × 10^6^ cells per well in 6-well plates, followed by pre-treatments with phospholipids and CORT insult. Then, cells were homogenised in lysis buffer and transferred into the filter tubes provided in the kit. Following procedure was performed according to manufacturer instructions. RNA concentration was determined using the ND-1000 spectrophotometer, and reverse transcription was assessed using the ExiLERATE LNA™ qPCR, cDNA synthesis kit (Exiqon). Subsequently, PCR reaction was performed using the ExiLERATE LNA™ qPCR, SYBR® Green master mix kit (Exiqon) in a lightcycler 480 II (Roche). Each sample was analysed in triplicate for both target gene and reference gene (β-actin), and the relative mRNA expressions were calculated using the 2^−ΔΔCt^ method (Livak and Schmittgen, 2001).

### 2.10 Statistical analysis

Statistical analysis was performed using the software SPSS 24.0, and the results were presented as mean ± SEM. Data from the neuroprotection experiment were analysed using the *t*-test to compare ‘Vehicle’ vs ‘CORT’; then the effect of ‘Phospholipids + CORT’ groups was compared to ‘CORT’ using one-way ANOVA followed by Dunnett's test. For proliferation studies, the data were analysed using two-way ANOVA followed by Tukey (HSD) post-test. For the differentiation experiment data, ‘Phospholipids’ groups were compared to ‘Vehicle’ using one-way ANOVA followed by Dunnett's test. A *p*-value of 0.05 was considered statistically significant.

## 3 Results

### 3.1 Phosphatidylserine, phosphatidylglycerol, phosphatidylethanolamine and phosphatidic acid attenuate CORT-induced cytotoxicity in cortical cells

**Fig. 2:**
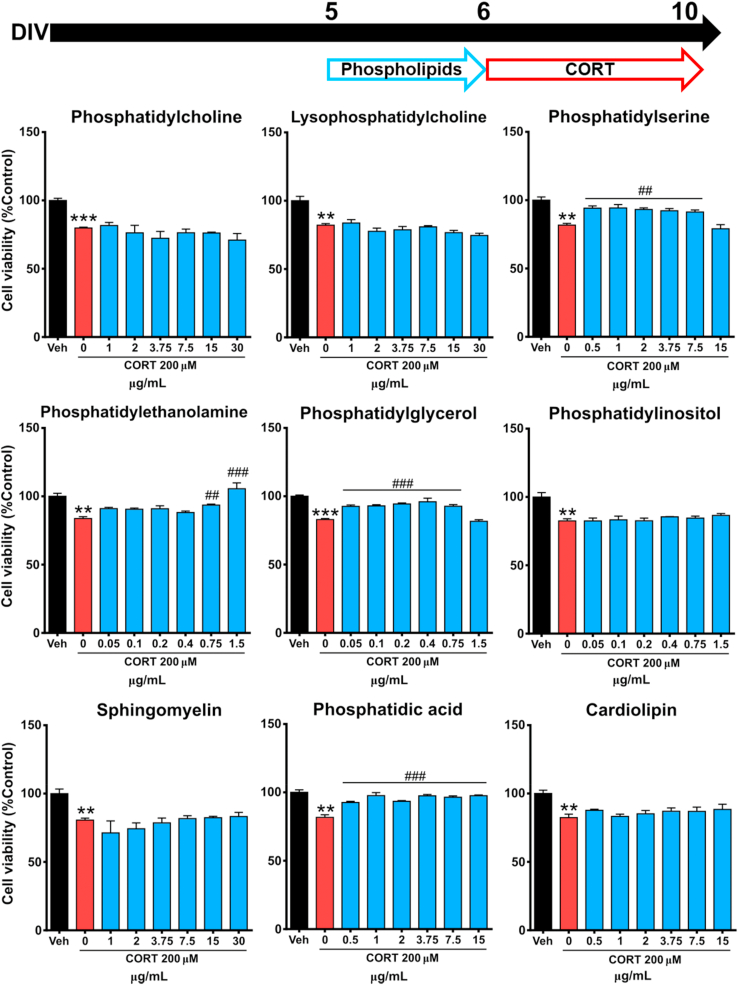
**Some phospholipids protect against CORT-induced cytotoxicity in cortical cells**. Cortical cells were pre-treated with the indicated concentrations of phospholipids for 24 h and then with 200 μM CORT for 96 h. Cell viability was measured by MTT assay. Results are expressed as the mean ± SEM of three independent experiments performed in triplicate (**p < 0.01; ***p < 0.001 versus ‘vehicle’ groups; ##p < 0.01; ###p < 0.001 versus ‘CORT’ groups).

To test the potential capacity of phospholipids to protect cortical cells from CORT-elicited cytotoxicity, cortical cells were first incubated for 24 h with different concentrations of each phospholipid to examine potential cytotoxicity (Fig. S1). None of the phospholipids tested in this study reduced cell viability in the range of concentration examined. Subsequently, cortical cells were pre-incubated for 24 h with phospholipids at different concentrations after 5 DIV, and then exposed to CORT for 96 h after removal of the phospholipid . A protective effect of phospholipid exposure was observed, specifically PS (0.5–7.5 μg/mL), PE (0.75–1.50 μg/mL), PG (0.05–0.75 μg/mL) and PA (0.5–15.0 μg/mL) were able to significantly reduce the cytotoxicity caused by 200 μM CORT exposure (Fig. 2).

### 3.2 Phosphatidylserine, phosphatidylglycerol and phosphatidylethanolamine inhibit CORT-induced alterations in neurons but not astrocytes

**Fig. 3:**
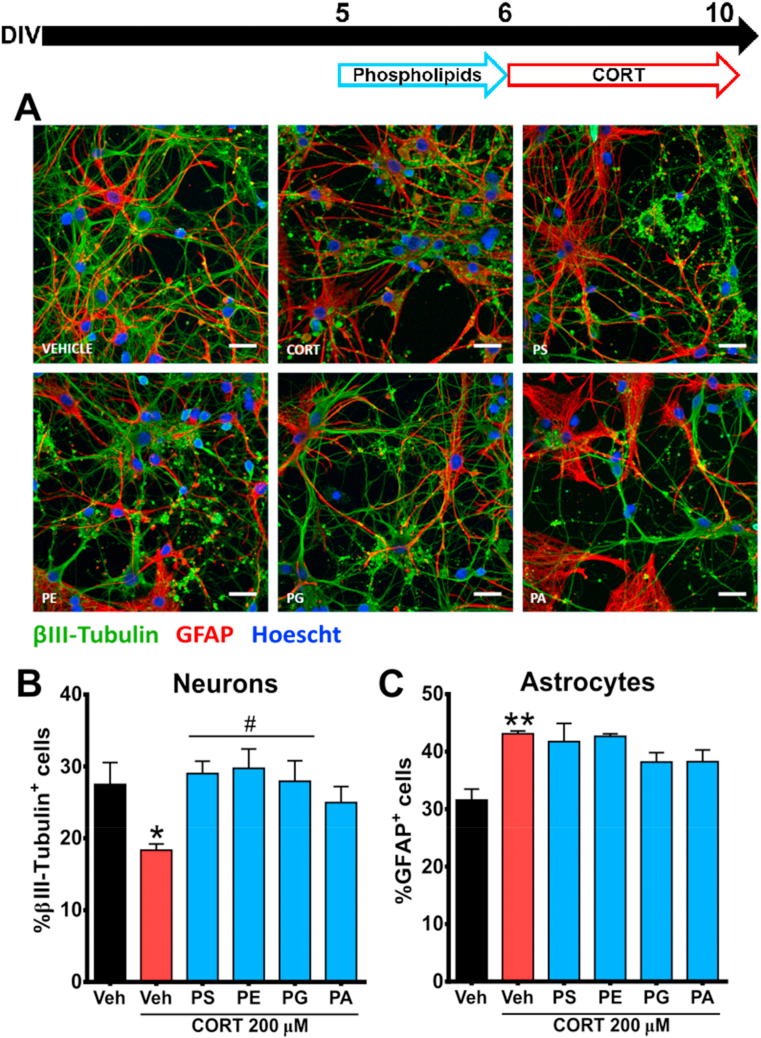
**Some phospholipids attenuateCORT-induced changes in neuron but not in astrocyte proportion**. **(A**–**C)** Cortical cells were pre-treated with phospholipids that showed protective effects in cell viability, including PS (4 μg/mL), PE (1.5 μg/mL), PG (0.4 μg/mL) and PA (1 μg/mL), and then exposed to CORT. Quantitative analysis of neurons and astrocytes was assessed by immunostaining of βIII-tubulin^+^ and GFAP^+^ cells. Scale bar = 50 μm. Results are expressed as the mean ± SEM of three independent experiments performed in triplicate (*p < 0.05; **p < 0.01 versus ‘vehicle’ groups; #p < 0.05 versus ‘CORT’ groups). Phosphatidylserine = PS; Phosphatidylethanolamine = PE; Phosphatidylglycerol = PG; Phosphatidic acid = PA.

To further determine the protective effects of PS (4 μg/mL), PE (1.5 μg/mL), PG (0.4 μg/mL) and PA (1 μg/mL) against CORT-induced cytotoxicity in cortical cells, we studied whether the negative impact of CORT on neuronal and astrocytic quantity and morphology (Pusceddu et al., 2016; Donoso et al., 2019) could be ameliorated by pre-incubation with these phospholipids. Concentrations for these phospholipids were based considering the protective range observed in the cell viability assay (Fig. 2). Indeed, 96-h exposure with CORT induced a significant reduction in the proportion of βIII-tubulin^+^ cells (neurons), from 27.49 ± 3.05% to 18.33 ± 0.88%; while the proportion of GFAP^+^ cells (astrocytes) was significantly increased from 31.56 ± 1.90% to 43.10 ± 0.49%. Interestingly, a 24-h pre-treatment with PS, PE and PG ameliorated the negative effects of CORT insult on neuronal integrity, as measured by an increase in proportion of βIII-tubulin^+^ cells (Fig. 3A). In addition, these phospholipids significantly abolished the CORT-induced reduction of βIII-tubulin^+^ cells (Fig. 3B). However, none of the selected phospholipids was able to reverse the CORT-induced increase of GFAP^+^ in the primary cultured cortical cells (Fig. 3C), suggesting this a secondary event following neuronal injury.

### 3.3 No effect of phospholipids on CORT-induced reduction of *Bdnf* expression in cortical cells

**Fig. 4:**
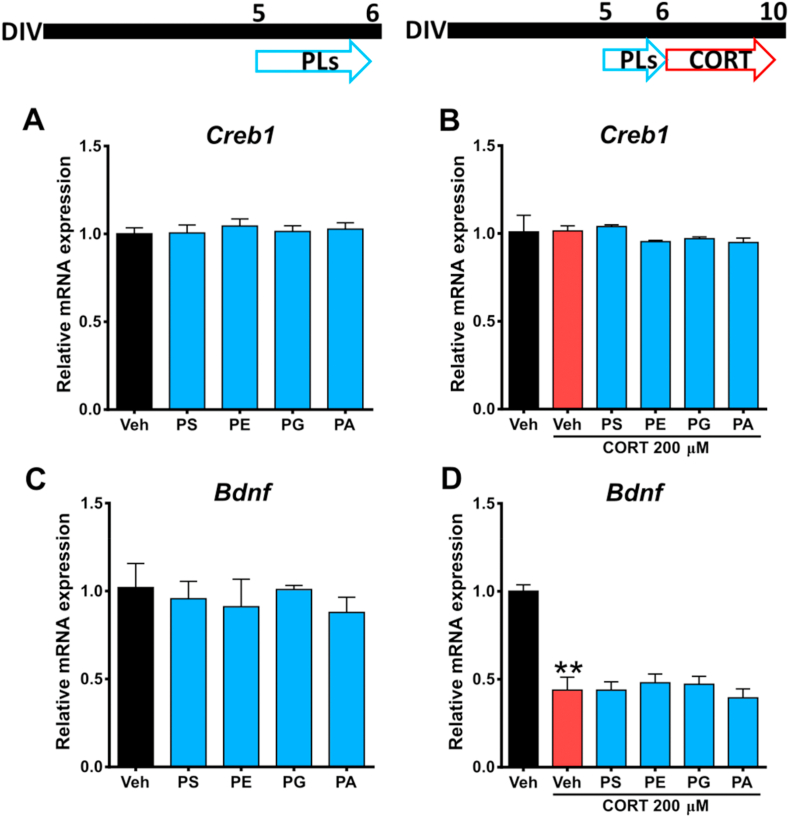
**Neuroprotective effects of phospholipids are not associated with Bdnf expression**. Total RNA was isolated under two different conditions; **(A & C)** Cortical cells were incubated with phospholipids for 24 h, then Bdnf and Creb1 expression was analysed. **(B & D)** Cortical cells were pre-incubated with phospholipids and then exposed to 200 μM CORT for 96 h. The gene expression was quantitatively measured using real time RT-PCR. Results are expressed as the mean ± SEM of three independent experiments performed in triplicate. (**p < 0.01 versus ‘vehicle’ groups). Phosphatidylserine = PS; Phosphatidylethanolamine = PE; Phosphatidylglycerol = PG; Phosphatidic acid = PA.

Expression of BDNF is highly associated with neuronal survival, and has been shown to be a mechanism of action of several nutritional components (Zhao et al., 2017; Shen et al., 2018; Srivastava et al., 2018; Khairy and Attia, 2019). Thus, we hypothesised that the phospholipid-mediated protective effect against CORT-induced neuronal injury may occur via this mechanism. Therefore, we explored the effects of PS, PE, PG and PA on CORT-induced decreased of *Bdnf* mRNA expression in cortical cells. In addition, we measured the mRNA levels of *Creb1*, an important nuclear factor involved in the expression of BDNF (Lee et al., 2009). However, 24-h incubations with PS, PE, PG and PA did not induce changes in the expression of *Creb1* (Fig. 4A), and its mRNA levels were unaffected after CORT exposure (Fig. 4B), suggesting that neither these phospholipids nor CORT are capable of modulating the upstream BDNF signalling pathway in terms of *Creb1* expression in cortical cells. In addition, a 24-h exposure to phospholipids did not produce significant changes in *Bdnf* expression (Fig. 4C). As expected, the 200 μM CORT significantly altered the expression of *Bdnf* by reducing its mRNA levels to 43.84 ± 7.33% compared to the vehicle. Nevertheless, no phospholipid treatment was able to ameliorate the *Bdnf* mRNA depletion following CORT insult, suggesting that the observed effect of these phospholipids is mediated by different mechanisms (Fig. 4D).

**Fig. 5:**
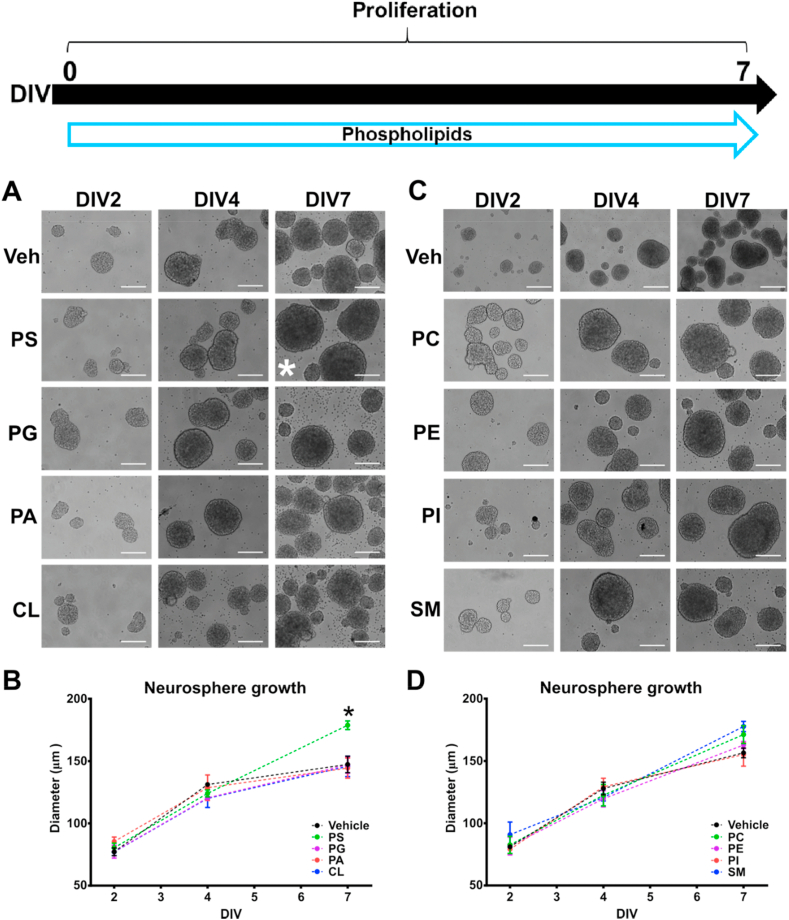
**Phosphatidylserine increases neurosphere growth in hippocampal NPCs.** Selected doses of phospholipids were incubated with NPCs cultures from DIV0 to DIV7; **(A**–**B)** NPC exposure to PS (4 μg/mL), PG (0.05 μg/mL), PA (4 μg/mL) and CL (1 μg/mL); **(C**–**D)** NPC exposure to PC (4 μg/mL), PE (0.75 μg/mL), PI (1.5 μg/mL) and SM (30 μg/mL). Quantitative analysis of NPC growth size was assessed by measuring neurosphere diameter. Scale bar = 100 μm. Results are expressed as the mean ± SEM of three independent experiments performed in triplicate (*p < 0.05 vs Vehicle group at DIV7). Phosphatidylcholine = PC; Phosphatidylserine = PS; Phosphatidylethanolamine = PE; Phosphatidylglycerol = PG; Phosphatidylinositol = PI; Sphingomyelin = SM; Phosphatidic acid = PA; Cardiolipin = CL.

### 3.4 Phosphatidylserine enhances proliferation of hippocampal neural progenitor cells

Next, we investigated whether phospholipids can modulate proliferation of hippocampal NPCs by inducing changes in the size of neurospheres. Neurosphere growth is associated with proliferative capacity, therefore to measure neurosphere size is a reliable method to visualise the effects of drugs and biochemical agents on proliferation (Bender et al., 2017; O'Leime et al., 2018; Yu et al., 2018). Analysis of untreated NPC neurosphere diameter revealed an increase over 7 days of around 100–120%, which it was considered to be strictly dependent of culture conditions (Crampton et al., 2012; Bender et al., 2017) (Fig. 5). On the other hand, treatment with PG (0.05 μg/mL), PA (4 μg/mL), CL (1 μg/mL), PC (4 μg/mL), PE (0.75 μg/mL), PI (1.5 μg/mL), and SM (30 μg/mL) did not alter the size of NPC neurospheres after a 7-day exposure (Fig. 5B and D). Doses of phospholipids were based in the maximum concentration that is not cytotoxic for neurosphere cultures after 7 DIV (data not shown). Notably, exposure to 4 μg/mL PS induced a significant increase of the neurosphere diameter at DIV7 by 14% compared to the control group, suggesting a positive effect on NPCs proliferation (Fig. 5B). LPC was excluded of this experiment as it demonstrated cytotoxic effects on NPC when exposed from DIV0 (data not shown).

### 3.5 Phosphatidylethanolamine increases astrocytic differentiation in hippocampal neural progenitor cells

**Fig. 6:**
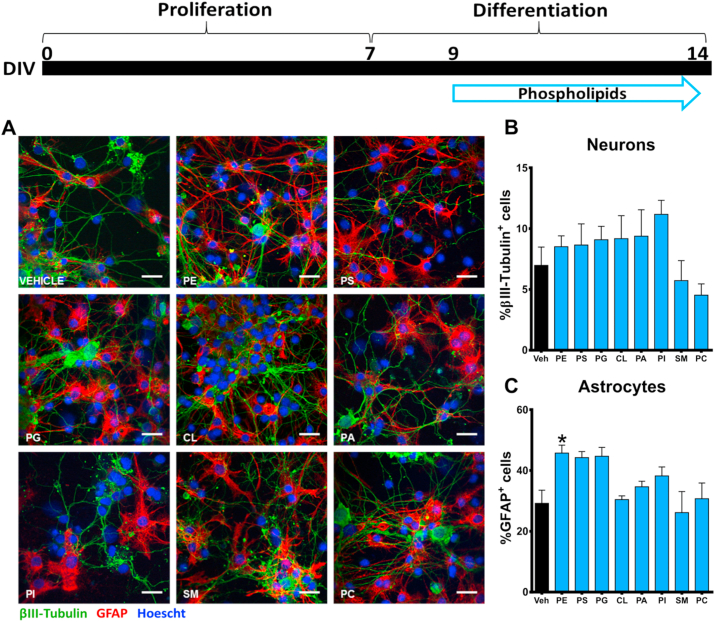
**Phosphatidylethanolamine increases the proportion of astrocytes in differentiated hippocampal NPCs. (A)** NPCs were incubated with selected doses of phospholipids in differentiation conditions for 5 days. **(B–C)** Quantitative analysis of neurons and astrocytes was assessed by immunostaining of βIII-tubulin^+^ and GFAP^+^ cells. Scale bar = 50 μm. Results are expressed as the mean ± SEM of three independent experiments performed in triplicate (*p < 0.05 versus ‘vehicle’ groups). Phosphatidylcholine = PC; Phosphatidylserine = PS; Phosphatidylethanolamine = PE; Phosphatidylglycerol = PG; Phosphatidylinositol = PI; Sphingomyelin = SM; Phosphatidic acid = PA; Cardiolipin = CL.

To explore the role of phospholipids on neural differentiation, we examined the proportion of astrocytes and neurons in NPCs following incubation with PC (4 μg/mL), PE (0.75 μg/mL), PI (1.5 μg/mL), SM (30 μg/mL), PS (4 μg/mL), PG (0.05 μg/mL), PA (4 μg/mL) and CL (1 μg/mL) (Fig. 6A). After a 5-day phospholipid exposure, no changes were detected in the proportion of βIII-tubulin^+^ cells (Fig. 6B). However, only PE demonstrated to be able to significantly increase the GFAP^+^ cell proportion.

## 4 Discussion

Phospholipids are structural lipids with recognised potential to protect neuronal functioning linked with cognition and behaviour, from the negative effects of aging and stress (Crook et al., 1991; Benton et al., 2001; Kullenberg et al., 2012; Boyle et al., 2019; Schverer et al., 2020). In the present study, we demonstrated that specific phospholipids have potential to significantly modify processes critically regulated under stress *in vitro*. In particular, we showed that PS, PE, PG and PA protect against CORT-elicited cytotoxicity in cortical cells and altered neuronal/astrocytic ratio. In addition, we demonstrated that PS facilitates neurosphere self-renewal, while PE improved astrocytic differentiation in hippocampal NPCs. Thus, our findings suggest a promising protective and beneficial effect of specific phospholipids in neuronal function *in vitro*.

Elevated concentration of CORT, the main stress hormone in rodent models, has been associated with depressive- and anxiety-like behaviours (Rosa et al., 2014; Mendez-David et al., 2017); and accumulating lines of evidence indicate that depressive or chronically stressed individuals have an over activated HPA axis, which ultimately is involved in the release of CORT to the bloodstream (Pariante and Lightman, 2008; Keller et al., 2017). Indeed, several *in vitro* studies showed that chronic exposure to CORT exerts toxic effects on neurons (Gao et al., 2015; Pusceddu et al., 2016; Zhao et al., 2018). In this regard, previous studies have suggested that phospholipid-enriched diets may possess therapeutic effects against stress-related conditions (Hellhammer et al., 2004; Boyle et al., 2019; Schverer et al., 2020). Therefore, evidence demonstrating that phospholipids prevent the neurotoxic effects of glucocorticoids *in vitro* might provide novel insights into the mechanism underpinning anti-stress effects *in vivo*. Our data show that CORT treatment decreased the cell viability of primary cultured cortical cells, in line with previous data from our lab and others (Pusceddu et al., 2016; Noh et al., 2018; Donoso et al., 2019; Shi et al., 2019). Next, we demonstrated that treatment with PS, PE, PG and PA significantly reduced this CORT-elicited cytotoxicity. However, we did not find a protective effect on CORT-induced cytotoxicity when using the phospholipids PC, LPC, PI, SM, and CL, indicating that not all phospholipids will possess this intrinsic beneficial effect.

The reason why other phospholipids tested did not exhibit this anti-stress effect *in vitro* may be associated to structural differences between these molecules, as similarly occurs in other physiological contexts. For example, it has been demonstrated that the anti-inflammatory properties of phospholipids strongly depend on their structure (Furnkranz and Leitinger, 2004; Lordan et al., 2017). Indeed, most of the phospholipids tested share similar chemical structure in the hydrophobic fatty acid tail, but their hydrophilic phosphate head differs from each other. PC is linked with choline, a molecule with demonstrated neuropharmacological potential (Kusuda et al., 2019), considering that the central cholinergic system is critically involved in memory (Blake et al., 2014), stress response (Mineur et al., 2013) and neurodevelopment (Bernhard et al., 2019). In contrast, PS has a serine attached to its carbon chain, an amino acid with proven capacity to modulate neuronal function (Billard, 2008). Thus, the neuroprotective capacity of PS, PE, PG and PA against CORT exposure might be related to their chemical structure in the hydrophilic head. Future studies are needed to demonstrate this structure-dependent function.

In addition, we confirmed the neuroprotective potential of PS, PE and PG by preventing the reduction of neuronal percentage in cortical cells caused by CORT treatment. None of the selected phospholipids were able to prevent the CORT-induced increase of astrocytes suggesting a phenotype-specific effect favouring neuronal viability. However, astrocytic overgrowth or astrogliosis is described as a critical process for neural protection and repair (Zhang et al., 2007), and it has been detected in similar studies using high exposure of CORT in neural tissue (Bridges et al., 2008; Pusceddu et al., 2016; Donoso et al., 2019). It is likely that the increase in astrocytes is a direct consequence to compensate CORT-induced neuronal injury and not a direct effect of phospholipid exposure.

To further investigate the potential mechanism underpinning the neuroprotective effect of PS, PE, PG and PA against CORT-elicited cytotoxicity we explored the implication of *Bdnf* gene expression. BDNF is a member of the neurotrophin family of proteins involved in neuroplasticity and neuronal survival (Brunoni et al., 2008), which also has been associated with neuroprotective effects *in vitro* (Almeida et al., 2005). In addition, patients suffering from stress-related disorders display decreased levels of BDNF, and its expression has strongly been implicated in antidepressant activity (Sen et al., 2008; Lee and Kim, 2010). We demonstrated that CORT induced a significant reduction of *Bdnf* mRNA levels, however none of the phospholipids were capable of preventing this negative consequence, suggesting that *Bdnf* rescue is not crucial for phospholipid-mediated anti-stress effect. In contrast, we did not find differences in *Creb1* mRNA levels after neither CORT nor phospholipids exposure. As CREB1 is a key nuclear factor involved in the expression of BDNF (Murphy et al., 2013), this result suggests that CORT-induced reduction expression of *Bdnf* mRNA is not dependent of *Creb1* expression.

Since there are not many pharmacological targets for phospholipids in neural tissue, we suspect that potential players involved in the phospholipid-mediated neuroprotection *in vitro* against CORT could belong to the Akt pathway. Indeed, one of the most important modulators of this signalling pathway is phosphoinositide 3-kinase (PI3K) (Hemmings and Restuccia, 2012), which actually has been found to be sensitive to some phospholipids (Yalcin et al., 2010; McNamara and Degterev, 2011). Future investigations are needed to elucidate the potential implications of the Akt pathway or other molecular systems in the neuroprotective mechanism of PS, PE, PG and PA.

Neurogenesis is another key brain process highly associated with mental health and cognition (Eisch et al., 2008), and highly vulnerable to stress (Schoenfeld et al., 2017). In order to investigate the effects of phospholipids on neuromodulatory processes associated with neurogenesis and neurodevelopment, we used hippocampal NPC cultures to evaluate proliferation and differentiation *in vitro*, thus providing new evidence highlighting a role for phospholipids in neuronal processes impacted under stress exposure. In this study, we found increased size of neurosphere diameter after treatment with PS. Neurosphere growth is a common measure of cell self-renewal and proliferation (Molofsky et al., 2003). Indeed, an increased in the neurosphere size is associated with proliferative activity of NPCs (O'Leime et al., 2018). Interestingly, PC, LPC, PI, PG, PA, PE, SM, and CL did not produce comparable effects to PS, suggesting a specific activity for this compound.

Our data revealed that only PE has a significant impact on hippocampal NPC astrocytic differentiation. Increased astrocyte differentiation has been associated with maintenance of adult neurons in terms of neurite growth and synaptic formation (Yasui et al., 2017). For example, high levels of synaptic glutamate can cause over-activation of neurons leading to excitotoxicity, and astrocytes have specific Na^+^ dependent transporters able to remove rapidly extracellular glutamate from the synaptic cleft, improving neuronal survival (Dong et al., 2009). Astrogenesis, the process where new astrocytes are produced, is mainly initiated by the activation of JAK-STAT, the canonical pathway regulating astrocyte gene expression (Bonni et al., 1997), and with important implications for stress-related disorders (Shariq et al., 2018). Nevertheless, the mechanism of phospholipid-mediated astrocytic differentiation in NPCs and the role of JAK-STAT activation herein remains to be investigated.

Considering that PS, the phospholipid tested in this study with positive effects on CORT-induced cytotoxicity and NPC proliferation, crosses the blood-brain barrier (BBB) and it is efficiently absorbed by neurons (Glade and Smith, 2015), it shows potential implications for future pre-clinical and clinical interventions involving stress-associated paradigms. To date, the capacity of other phospholipids to cross the BBB has not been confirmed yet. However, since the chemical and physical properties of the BBB allow the transport of a number of molecules with specific features (Fong, 2015), including drugs and chemicals with zwitterionic structure, it is plausible that most of the phospholipids tested in our work are capable of crossing it.

Given the importance of PC and choline for the CNS, including critical implications for some neurobiological processes such as memory (Bekdash, 2019), cognition (Li et al., 2020) and neurodevelopment (Bernhard et al., 2019), we believe that the lack of positive effects of PC in our cellular models may be interpreted as a dependence of a complete physiological context to exert its neurobiological effects. Indeed, many neuroactive chemicals need previous systemic metabolism to induce important changes in the brain and behaviour (Hung et al., 2012; Mardal et al., 2016). Future pre-clinical research could address the implications of systemic metabolism of phospholipids in their potential mental health benefits.

In this work we evaluated the neurobiological effects of individual phospholipids in neuronal *in vitro* models. Nevertheless, different dietary sources of phospholipids may contain several types of phospholipids that can be simultaneously incorporated into the host metabolism (Kullenberg et al., 2012), suggesting that these molecules could exert their health benefits in combination. In addition, natural sources of phospholipids also may include Omega-3 fatty acids (Burri et al., 2012), such as docosahexaenoic acid (DHA) and eicosapentaenoic acid (EPA), as well as choline (Smolders et al., 2019), which are considered as important neuromodulatory compounds (Bjelland et al., 2009; Abdolahi et al., 2019). We consider that the screening of different mixtures of phospholipids and direct natural sources of them would be an interesting and physiologically relevant area of future research.

In conclusion, our present work confirmed that specific phospholipids have the ability to modulate important neuronal functions, such as neuroprotection and neurodevelopment which are impacted following the stress response. To our knowledge, this is the first study screening a wide range of individual phospholipids in neuronal *in vitro* models. In particular, we demonstrated that PS, PE, PG and PA protected cortical neurons against CORT-induced neurotoxicity. In addition, we found that PS produced a significant increase in hippocampal NPC neurosphere size, which is associated with active neuronal proliferation. Finally, we detected that astrocytic differentiation on hippocampal NPCs was improved after treatment with PE. Our findings support that specific phospholipids may have potential beneficial effects for brain health and a protective capacity against stress-related mental disorders (Kullenberg et al., 2012; Boyle et al., 2019; Schverer et al., 2020), where these effects could be partly mediated by modulation of neuronal/astrocytic plasticity. Although the signalling pathways involved in the phospholipid-mediated neurobiological effects, and their capacity to cross the blood-brain-barrier need to be further explored, we provide novel insights into the potential role of phospholipids on the CNS. Future pre-clinical studies based in phospholipid-enriched supplementation in animal models of stress should address the potential implication of these compounds on behaviour and neuroplasticity.

## Funding

This work was supported by 10.13039/501100001602Science Foundation Ireland in the form of a Research Centre grant (SFI/12/RC/2273) to APC Microbiome Ireland and by a research grant from Cremo S.A.

## CRediT authorship contribution statement

**Francisco Donoso:** Investigation, Writing - original draft, Visualization, Formal analysis. **Marina Schverer:** Validation, Investigation, Writing - review & editing. **Kieran Rea:** Supervision, Project administration, Resources. **Matteo M. Pusceddu:** Methodology. **Bernard L. Roy:** Conceptualization, Funding acquisition. **Timothy G. Dinan:** Supervision, Conceptualization. **John F. Cryan:** Supervision, Conceptualization, Writing - review & editing. **Harriët Schellekens:** Supervision, Conceptualization, Writing - review & editing.

## Declaration of competing interest

APC Microbiome Ireland has conducted studies in collaboration with several companies, including GSK, Pfzer, Cremo, Suntory, Wyeth, Mead Johnson, Nutricia, 4D Pharma, and DuPont. B. L. Roy is an employee of Cremo SA. T. G. Dinan has been an invited speaker at meetings organized by Servier, Lundbeck, Janssen, and AstraZeneca and has received research funding from Mead Johnson, Cremo, Nutricia, and 4D Pharma. J. F. Cryan has been an invited speaker at meetings organized by Mead Johnson, Yakult, Alkermes, and Janssen and has received research funding from Mead Johnson Nutrition, Cremo, Nutricia, DuPont, and 4D Pharma.
