# Supplementary material for: Neurobiological effects of phospholipids in vitro: Relevance to stress-related disorders
Source: Neurobiol Stress. 2020 Sep 15;13:100252. doi: 10.1016/j.ynstr.2020.100252 (PMC7739190; doi:10.1016/j.ynstr.2020.100252)
Supplement: Multimedia component 1 [file mmc1.docx]

**Supplementary information**


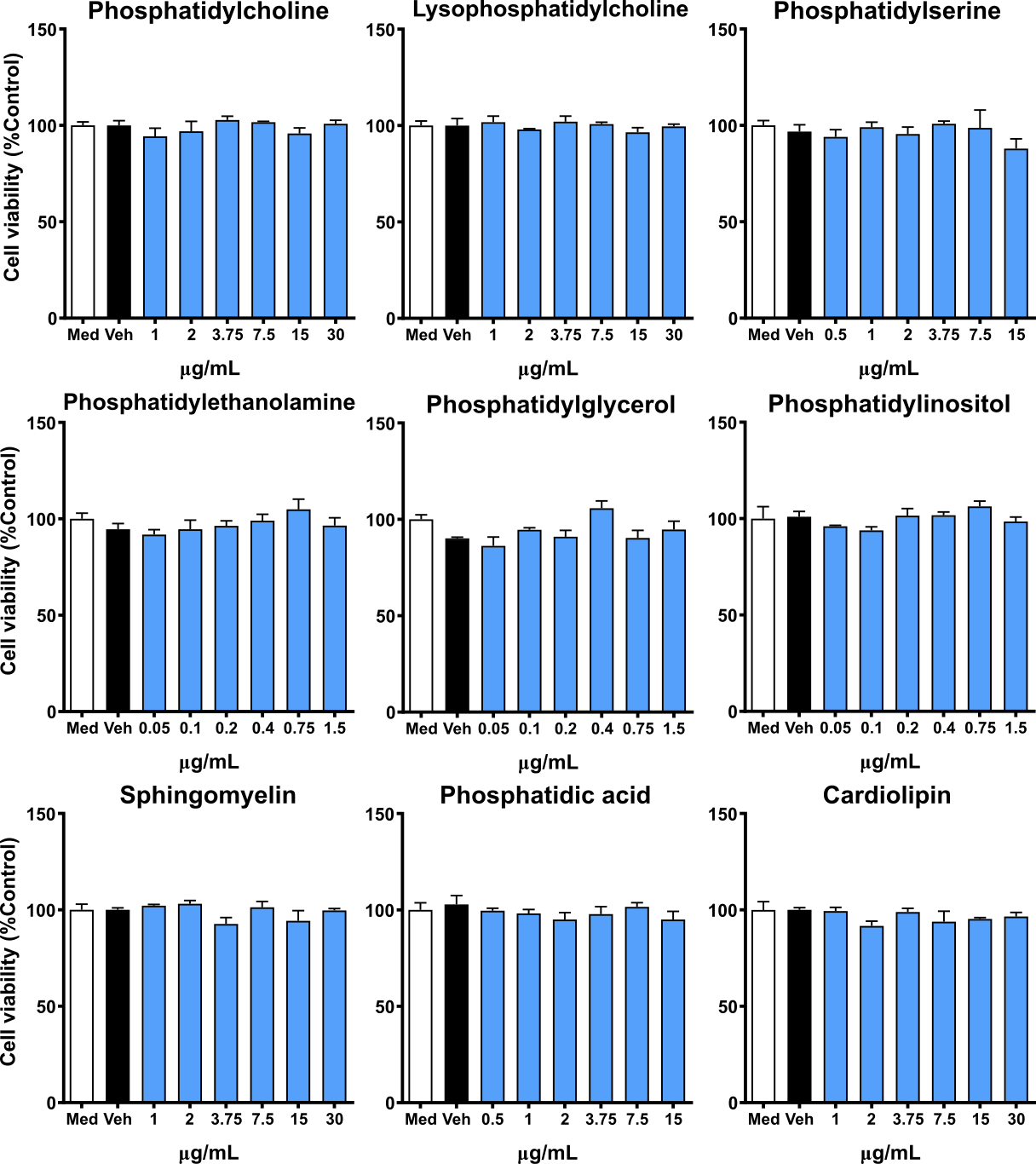


***Figure S1 Phospholipids do not exert cytotoxic effects on cortical cells****. Cortical cells were incubated with various concentrations of phospholipids for 24 hours. Different concentration ranges were tested for each phospholipid considering their solubility. Cell viability was measured by MTT assay. Results are expressed as the mean ± SEM of three independent experiments performed in triplicate. Medium = Med; Vehicle = Veh*
